# Cysteine Leukotriene Receptor Antagonist—Montelukast—Treatment Improves Experimental Abdominal Aortic Aneurysms in Mice

**DOI:** 10.1155/2024/8826287

**Published:** 2024-11-27

**Authors:** Kexin Li, Meng Li, Panpan Wei, Kangli Tian, Haole Liu, Weilai Fu, Haiwen Hou, Yajie Chen, Baohui Xu, Yankui Li, Sihai Zhao

**Affiliations:** ^1^Institute of Cardiovascular Science, Translational Medicine Institute, Xi'an Jiaotong University Health Science Center, Xi'an, Shaanxi 710061, China; ^2^Department of Vascular Surgery, The Second Hospital of Tianjin Medical University, Tianjin 300211, China; ^3^Department of Pathology, School of Basic Medicine, Luoyang Polytechnic College, Luoyang, Henan 471000, China; ^4^Guangdong Provincial Key Laboratory of Large Animal Models for Biomedicine, School of Pharmacy and Food Engineering, Wuyi University, Jiangmen, Guangdong 529000, China; ^5^Department of Surgery, Stanford University School of Medicine, Stanford, California 94305, USA

**Keywords:** abdominal aortic aneurysm, elastin, inflammation, montelukast, smooth muscle cells

## Abstract

**Background:** Cysteinyl leukotrienes (LTs) and their receptors are involved in the pathogenesis of abdominal aortic aneurysms (AAAs). However, whether CysLT1 receptor antagonists such as montelukast can influence experimental nondissecting AAA remains unclear.

**Methods:** Nondissecting AAAs were induced in C57BL/6J mice by transient aortic luminal infusion of porcine pancreatic elastase (PPE). All animals were administrated montelukast (1 or 10 mg/kg, daily) or vehicle by gavage beginning 1 day before PPE infusion for 14 days. On day 0 (baseline) and day 14 after PPE infusion, abdominal aortic diameters were directly measured. Aortic aneurysmal segment samples were collected, and histopathological analysis was performed.

**Results:** Compared to vehicle treatment, montelukast significantly decreased PPE infusion–induced aortic expansion in a dose-dependent manner (0.09-mm reduction at a low dose and 0.19-mm reduction at a high dose). Histopathological analysis also revealed that compared with vehicle treatment, montelukast treatment, especially in the high-dose group, significantly improved PPE-induced aortic elastin degradation and medial smooth muscle cell depletion. Both doses of montelukast also markedly decreased the number of local leucocytes, including macrophages, CD4^+^ T cells, CD8^+^ T cells, and B cells, infiltration and accumulation in aortic aneurysmal lesions. Montelukast treatment also downregulated matrix metalloproteinase 2 (MMP2) and MMP9 expression and inhibited mural angiogenesis in aneurysmal aortas.

**Conclusion:** Montelukast treatment improves experimental nondissected AAAs in mice partly by improving aortic inflammation.

## 1. Introduction

Abdominal aortic aneurysm (AAA) is characterized by irreversible expansion of the abdominal aorta that exceeds 50% of the normal diameter of the abdominal aorta due to various cardiovascular risk factors [1–3]. The development of AAA is occult, and there are usually no obvious symptoms in the early stages. Many AAA patients are diagnosed with an increase in the diameter of the abdominal aorta during physical examinations. The mortality rate of sudden aneurysm rupture can reach over 80%, and many patients will die shortly after admission to the hospital [1, 4, 5]. At present, AAA has become a serious disease that affects people's health. Currently, no effective drug treatment is available for patients with aneurysms, and controlling risk factors and endovascular aneurysm repair (EVAR) are the main methods used for AAA management [4, 5].

The pathogenesis of AAA is often thought to be the result of a combination of genetic and environmental factors, including age, sex, smoking status, race, chronic inflammation, hypertension, and atherosclerosis [3, 6]. Several pathological mechanisms have been involved in AAA formation, including vascular extracellular matrix remodeling, inflammatory and immune responses, oxidative stress, and dysfunction of vascular smooth muscle cells (SMCs) [7]. Despite the increasing progress in our knowledge about the etiopathology of AAA, an effective pharmacological treatment against high-risk or small aneurysm patients is still unavailable [8]. Immune-mediated leucocyte infiltration and aortic wall destruction play significant roles in the pathogenesis of AAA [9–11]. Anti-inflammatory agents have been shown to inhibit the progression of aneurysms in experimental AAA animal models [5, 12–14]. There are several anti-inflammatory drugs in clinical practice whose pharmacology and side effects are relatively clear, and they are potential anti-AAA candidates. If confirmed to have anti-AAA properties, these drugs can be quickly applied in the clinic.

Cysteinyl leukotrienes (LTs) are potent inflammatory mediators involved in the pathogenesis of AAA [15]. Montelukast is an approved clinical antiasthma drug that has a high affinity and selectivity for CysLT1 receptors and can effectively inhibit the physiological effects of LTs binding to CysLT1 receptors without any receptor agonist activity. Montelukast inhibits angiotensin II (Ang II)–induced aortic dissection in mice [16]. Whether montelukast influences nondissecting aneurysms, such as in a porcine pancreatic elastase (PPE)–induced experimental AAA model, remains unclear. We hypothesized that montelukast may improve nondissecting AAAs by inhibiting aortic inflammation. These results indicate that montelukast inhibits experimental AAAs partly by reducing aortic leucocyte infiltration and mural angiogenesis and preserving medial elastin and SMCs.

## 2. Materials and Methods

### 2.1. Mice

Thirty male C57BL/6J mice (9–12 weeks old) were provided by the Laboratory Animal Center of Xi'an Jiaotong University and were used to induce AAAs in this study. The mice were housed in a university animal facility with a 12-h light/12-h dark cycle.

### 2.2. Generation of AAAs in Mice

Freshly prepared PPE (Cat #, E-1250; Sigma-Aldrich, St. Louis, United States, diluted to 1.5 U/mL with PBS before use) solution was used for abdominal aortic infusion to induce aneurysms. After the mouse was anesthetized with 2% isoflurane inhalation, an incision approximately 2~3 cm long was made along the midline of the mouse abdomen, which fully exposed the abdominal aorta, bluntly separated the perivascular tissue, separated the branch vessels, and was ligated to prevent leakage during PPE infusion. The surgical segments upstream and downstream of the abdominal aorta were controlled by slipknots, the abdominal aortic segment was punctured with 8-0 suture needles, the blood was quickly removed with cotton swabs, and a PE-10 cannula was inserted into the aorta through the opening. After the cannula was fixed via slipknot ligation, approximately 30 *μ*L of PPE was rapidly infused with a syringe pump, and after continuous infusion under pressure for 5 min, the cannula was removed, a cotton swab was used to remove the PPE residue, the aortotomy was sutured with 11-0 silk sutures, and the abdominal cavity was closed [17–19].

### 2.3. Montelukast Treatment

Montelukast for the animal experiments in this study was produced by Organon Pharma (United Kingdom) Limited (subpackaging manufacturer, Hangzhou MSD Pharmaceutical Co. Ltd.) and was freshly prepared in saline immediately prior to use. All mice were randomly divided into three groups. Montelukast at a low dose (1 mg/kg, daily, *n* = 10) or high dose (10 mg/kg, daily, *n* = 10) was given to the mice by oral gavage starting 1 day prior to PPE infusion for 14 days. Mice receiving an equal amount of saline served as vehicle controls (*n* = 10). The doses of montelukast used in the animal experiments were selected according to previous studies [16, 20, 21].

### 2.4. Measurements of Aortic Diameters

During surgery, infrarenal aortas prior to PPE infusion were photographed under a stereomicroscope, and aortic outer diameters were measured in situ with image software (Images Plus 3.0, Motic, Xiamen, China) as the baseline. At the end of the experiment, an overdose of inhaled carbon dioxide was used to euthanize the animals, and the diameter on day 14 after PPE infusion was measured as described above. An AAA is defined when the aortic dilation exceeds 50% of the baseline diameter [22].

### 2.5. Histopathological Experiments of AAA in Mice

After 14 days of PPE infusion, all mice were euthanized, the aortic aneurysmal segments were isolated and embedded in OCT, and frozen tissue blocks were serially sectioned (5 *μ*m) for histopathological staining. Hematoxylin and eosin (H&E) and elastic van Gieson (EVG) staining was conducted following previously published procedures [22, 23]. Immunohistochemical (IHC) staining with monoclonal/polyclonal antibodies (Abs) was performed by following the standard biotin–streptavidin peroxidase method [12]. Smooth muscle alpha actin (1:400; Cat #, 19245S; CST, Danvers, MA) IHC staining was performed to analyze aortic medial SMC loss after PPE infusion. To assess the inflammation status of the aortic aneurysmal segments, IHC staining of leucocyte subsets, including macrophages (CD68, 1:200; Cat #, 137002; BioLegend, San Diego, CA), CD4^+^ T cells (CD4, 1:200; Cat #, 100402; BioLegend), CD8^+^ T cells (CD8, 1:200; Cat #, 100702; BioLegend), and B cells (CD45R, 1:200; Cat #, 103202; BioLegend), was also conducted. For the PPE-induced angiogenesis analysis, a CD31 mAb (CD31, 1:200; Cat #, 102402; BioLegend) was used to mark the mural neovessels. Primary Abs against MMP2 (1:200; Cat #, AF1488, R&D Systems, Minneapolis, MN) and MMP9 (1:200; Cat #, AF909, R&D Systems) were also used to measure the expression levels of these two proteins involved in the pathogenesis of AAA. The biotinylated secondary Abs used in this study included an anti-rat Ab (1:400; Cat #, BA-9400; VECTOR, Burlingame, CA) and an anti-rabbit Ab (1:400; Cat #, BA-1000-1.5; VECTOR). Other reagents used were a streptavidin–peroxidase conjugate (1:400; Cat #, 016-030-084; Jackson ImmunoResearch Laboratories Inc., West Grove, PA) and an AEC substrate kit (Cat #, SK-4200, VECTOR).

### 2.6. Quantification and Semiquantification of Histological Staining

Semiquantification of medial elastin degradation was performed according to previously reported methods [18, 19, 22]. The histological score of elastic layer break/degradation was divided into four levels from mild to severe. In Grade I, the destruction of the elastic layer is limited to only one layer; in Grade II, the elastin degradation involves two or all layers but is limited to one-fourth of the vessels; in Grade III, the elastin degradation involves all elastic layers but less than one-half of the vessels; and in Grade IV, the elastin degenerates beyond three-fourths of the vessels. Similarly, medial SMC depletion and macrophage infiltration severity grading was also performed by following previously reported methods [22]. Other leucocyte subset quantifications were conducted by counting the cell numbers per aortic cross section (ACS). MMP2 and MMP9 expression was quantified by measuring the positively stained areas with image software (WinRoof 6.5, Mitani Co. Ltd., Tokyo, Japan). Aortic mural angiogenesis was evaluated by counting the number of CD31-positive stained neovessels.

### 2.7. Statistical Analysis

All quantitative data are expressed as the mean ± standard deviation (SD). The ordinal data (histological scores) are presented as medians and interquartile ranges. For normally distributed data, one-way or two-way ANOVA was used, followed by multiple comparisons for two group comparisons. Three unmatched groups were compared using the nonparametric Kruskal–Wallis test for normally distributed data. All the statistical analyses were performed with PRISM 9.0, and *p* < 0.05 was considered to indicate statistical significance.

## 3. Results

### 3.1. Montelukast Inhibits PPE-Induced Abdominal Aortic Dilation

**Figure 1:**
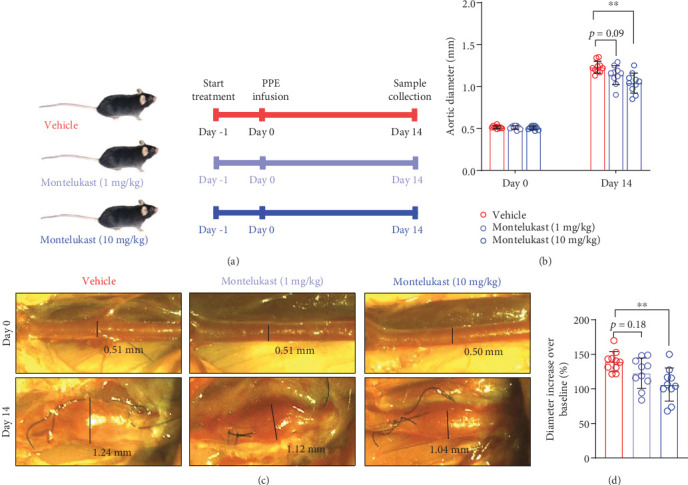
Montelukast inhibits the increase in aortic diameter in mice with experimental AAAs. (a) Experimental design: all mice received PPE infusion to model AAAs. Two doses of montelukast (1 or 10 mg/kg, daily) or vehicle were given by gavage (*n* = 10 each group). (b) Aortic diameters on Day 0 (baseline) and Day 14 (sample collection timepoint) after PPE infusion. Two-way ANOVA with multigroup comparisons was performed; ⁣^∗∗^*p* < 0.01. (c) Representative images of abdominal aortas on Day 0 and Day 14 after surgery. (d) Increase in aortic diameter over baseline (%). One-way ANOVA with comparisons between two groups was carried out (⁣^∗∗^*p* < 0.01).

To clarify the effects of montelukast on PPE-induced experimental AAAs, mice were administered montelukast (1 or 10 mg/kg) or vehicle by gavage (Figure 1(a)). The baseline aortic diameter (day 0) was similar among the three groups (Figures 1(b) and 1(c)). Compared to vehicle treatment, two doses of montelukast decreased the aortic diameter by 0.09 mm (1 mg/kg) and 0.19 mm (10 mg/kg) (Figure 1(b)). Both doses of montelukast tended to inhibit PPE infusion–induced aortic expansion; however, only high-dose treatment (10 mg/kg) significantly inhibited this effect (1.04 ± 0.11 vs. 1.23 ± 0.07 mm) (Figure 1(b)). The percentage increase in the diameter of the PPE infusion–induced aorta relative to the baseline diameter was also calculated. The changes in aortic diameter in mice treated with high-dose montelukast were significantly smaller than those in vehicle-treated mice (139.47 ± 13.85 vs. 106.14 ± 22.78%, respectively) (Figure 1(d)). These results suggest that montelukast might have an antianeurysm effect on mice with experimental AAAs.

### 3.2. Montelukast Improves Medial Elastin Destruction and SMC Depletion

**Figure 2:**
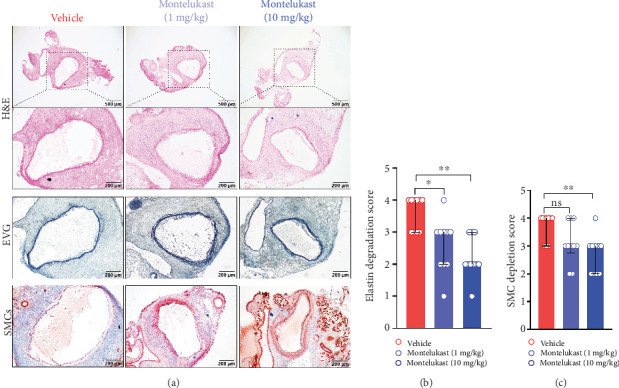
Montelukast inhibits PPE infusion–induced aortic medial elastin destruction and SMC depletion. (a) Representative images of histopathological staining of aneurysmal aortic segments in frozen sections. (b) Histological grading and semiquantification of aortic medial elastin (*n* = 10/group). (c) Histological grading and semiquantification of aortic medial SMC depletion (*n* = 10/group). One-way ANOVA with a multigroup nonparametric Kruskal–Wallis test was performed (⁣^∗^*p* < 0.05 and ⁣^∗∗^*p* < 0.01). NS, not significant.

Medial SMCs and elastin are very important for maintaining normal physiological function of the artery. H&E staining revealed that the abdominal aortas of PPE-infused mice had characteristics of AAA, such as a decrease in the number of SMCs, the disappearance of the lamellar structure of vascular SMCs, the destruction of the elastic layer, and inflammatory cell infiltration (Figure 2(a)). Compared with vehicle treatment, montelukast treatment significantly improved PPE-induced aortic elastin degradation. The aortic elastin histological scores were 4 (3–4, median with interquartile range) for the vehicle-treated mice, 3 (2–3) for the low-dose montelukast–treated mice, and 2 (2–3) for the high-dose montelukast–treated mice (Figures 2(a) and 2(b)). Similar to the elastin scores, high-dose montelukast treatment also significantly improved medial SMC loss compared to vehicle treatment, as shown by SMC IHC analysis (Figures 2(a) and 2(c)). The antianeurysmal effects of montelukast might be associated with protecting aortic medial elastin and SMCs.

### 3.3. Montelukast Inhibits Leukocyte Accumulation in Aneurysmal Lesions

**Figure 3:**
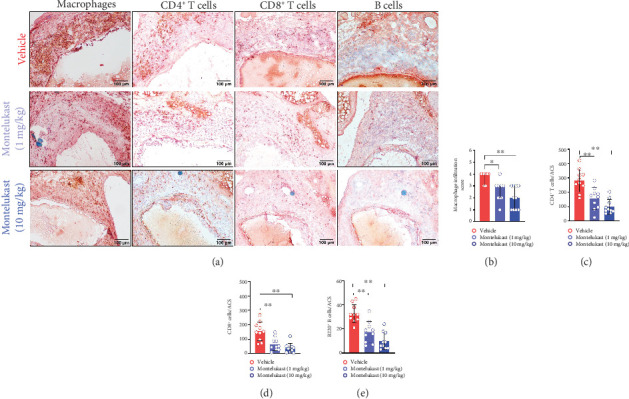
Montelukast decreases aortic leukocyte accumulation. (a) Representative images of aortic infiltrated leukocytes, macrophages, T cells, and B cells. (b–e) Quantification or semiquantification of leukocyte subsets per ACS (*n* = 10/group). For normally distributed data, one-way ANOVA was used (for T cells and B cells), and the nonparametric Kruskal–Wallis test was used for semiquantification of the macrophage score. ⁣^∗^*p* < 0.05 and ⁣^∗∗^*p* < 0.01.

An abnormal inflammatory response plays an important role in the development of AAA. Murally infiltrated macrophages, T cells, and B cells were detected by IHC staining. Diffuse infiltrated macrophages were predominant among the subsets of inflammatory cells in the aneurysmal aortic segment (Figure 3(a)). Both doses of montelukast (3 (2–3) at the low dose and 2 (2–3) at the high dose) markedly decreased macrophage infiltration compared with vehicle treatment (4 (3–4), especially at the high dose) (Figures 3(a) and 3(b)). Similar to its effect on macrophages, montelukast treatment also significantly reduced the accumulation of CD4^+^ T cells (282/ACS in vehicle, 156/ACS in low-dose montelukast, and 99/ACS in high-dose montelukast, respectively), CD8^+^ T cells (154/ACS in vehicle, 65/ACS in low-dose montelukast, and 38/ACS in high-dose montelukast, respectively), and B cells (33/ACS in vehicle, 17/ACS in low-dose montelukast, and 10/ACS in high-dose montelukast, respectively) in aneurysm lesions compared with vehicle treatment (Figures 3(c), 3(d), and 3(e)). These results showed that the inhibition of inflammation may partly mediate the antianeurysm effect of montelukast.

### 3.4. Montelukast Treatment Downregulates MMP2 and MMP9 Expression in Aneurysmal Lesions

**Figure 4:**
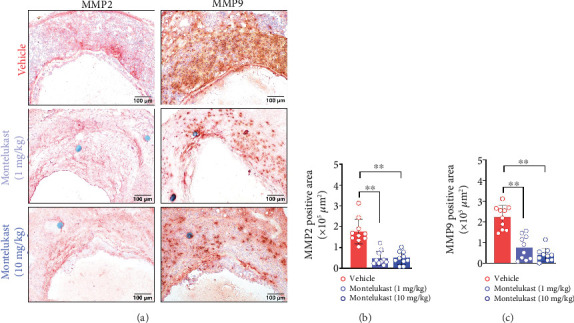
Montelukast downregulated MMP2 and MMP9 expression. (a) Representative IHC images of aortic MMP2 and MMP9. (b, c) Quantification of MMP2- and MMP9-positive areas in the aneurysmal aorta. One-way ANOVA was used (*n* = 10/group). ⁣^∗∗^*p* < 0.01.

Inflammatory cells produce MMPs, especially MMP2 and MMP9, which promote the progression of AAA. Compared with those in vehicle-treated mice, the expression levels of aortic MMP2 and MMP9 were also decreased in montelukast-treated mice (Figure 4). Montelukast treatment reduced the MMP2-positive area by approximately 72% (Figure 4(a)) and decreased the MMP9-positive area by 65% in the low-dose group and 82% in the high-dose group (Figure 4(b)).

### 3.5. Montelukast Treatment Reduces Mural Neovessel Numbers

**Figure 5:**
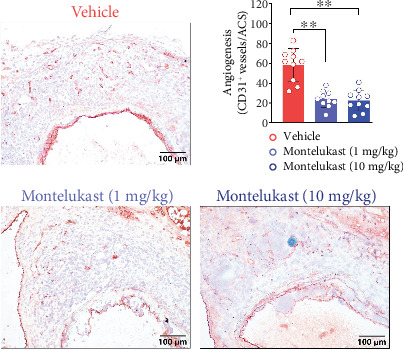
Montelukast treatment reduces mural neovessel numbers. Mural neovessels were marked by CD31 IHC staining, and angiogenesis was evaluated by counting the number of neovessels per ACS. One-way ANOVA was performed for statistical analysis (*n* = 10/group). ⁣^∗∗^*p* < 0.01.

Aortic adventitia angiogenesis accelerates the progression of AAA. On Day 14 after PPE infusion, the mural neovessel densities were 59/ACS in mice that received vehicle, 24/ACS in mice that received a low dose of montelukast, and 23/ACS in mice that received a high dose of PPE (Figure 5). Montelukast treatment significantly decreased the mural neovessel density in the aneurysmal aorta by approximately 60%.

## 4. Discussion

Due to the lack of effective drug theripes, EVAR remains the main treatment for clinical AAA [1, 2, 4, 24–28]. The pathogenesis of AAA has not been fully elucidated, which may have halted the development of antianeurysm drugs [24]. Except the classic mechanisms, such as inflammation, oxidative stress, and SMC depletion, microbiome-dependent metabolite trimethylamine N-oxide (TMAO) was also reported to be associated with AAA formation, and inhibition of TMAO may serve as a novel therapeutic approach for AAA treatment [29]. Genome-wide association studies and Mendelian randomization analyses have identified some novel drug targets, and several placebo-controlled randomized trials have tested potential drugs to slow AAA growth; however, none of these trials have shown convincing evidence of drug efficacy [24]. Aortic wall inflammation may play a central role in AAA pathogenesis [9–11, 30]. Many anti-inflammatory molecules have been tested in animal models to screen potential antianeurysm drugs [14, 31–33]. Montelukast is a drug used to control asthma and allergic rhinitis that can specifically inhibit CysLT1 receptors in the airways, thereby improving airway inflammation. It was also found that montelukast can significantly suppress proinflammatory mediator/chemokine expression and partly abolish the inflammatory response [34–36]. In this study, the anti-inflammatory property of montelukast was found to improve PPE-induced AAAs in mice.

The expression of LT C4 synthase is upregulated in the media and adventitia of the aortic aneurysmal wall and is localized in areas rich in infiltrated leucocytes [15, 20, 37]. Montelukast is the most prescribed CysLT1 antagonist used in asthmatic patients [38]. Animal studies reported that using a LT receptor antagonist brings to a reduction of cardiovascular disease risk, including acute coronary syndrome, myocardial infarction, ischemic stroke, or atherosclerosis [39, 40]. LTs have also been implicated in aneurysms and may act as potential targets for the treatment of AAA. Previous studies also reported that montelukast treatment suppressed Ang II-induced aortic dissection [16, 20]. In this study, montelukast treatment was found to limit PPE-induced aortic expansion in a dose-dependent manner in mice. PPE-induced nondissecting AAAs or Ang II-induced dissecting models contain false lumens that represent the two types of AAA animal models that are currently widely used [41]. In the present study, we first reported the protective effect of montelukast on the progression of nondissecting AAAs. Challenging aneurysmal tissue with exogenous LTs can increase the release of MMP2 and MMP9, and montelukast can block this effect [15]. MMP2 and MMP9 are key factors involved in the occurrence and development of AAA by degrading the extracellular matrix and elastin [42, 43]. In this in vivo study, both doses of montelukast reduced the expression of MMP2 and MMP9 in the murals, which is consistent with the findings of previous studies. These effects of montelukast on MMPs may also contribute to the preservation of medial elastin. The mice that received montelukast showed less aortic elastin degradation than did the vehicle-treated mice in this study. Montelukast decreases the expression of MMPs, possibly also related to its ability to inhibit aortic leucocyte infiltration, which is a main source of local MMPs and contributes to many cardiovascular diseases [44]. Montelukast can inhibit neutrophil proinflammatory activity by a cyclic AMP–dependent mechanism [45]. The inhibitory effect of montelukast on MMP2 and MMP9 expression may also be associated with a decreased number of leucocytes in the aneurysmal aortic segments, their level of MMP expression, or both [46]. In another way, montelukast may also inhibit MMP9 expression and secretion through other mechanism that might be independent of its antagonist effect on the CysLT1 receptor [47, 48]. These effects of montelukast on MMPs may also contribute to the preservation of medial elastin and inhibit aortic diameter expansion.

Montelukast was found to inhibit leukocyte migration from the systemic circulation into the alveoli in asthma models [49]. Among the targets of CysLT1R antagonists in allergic rhinitis are the vascular bed and infiltrating leukocytes, such as mast cells, eosinophils, and macrophages [50]. The massive amount of infiltrated leucocytes further contributes to the progression of AAA by producing proinflammatory molecules such as cytokines and MMPs. In this study, montelukast treatment also inhibited experimental AAAs by decreasing the infiltration of aortic leucocytes, including macrophages, CD4^+^ T cells, CD8^+^ T cells, and B cells. In addition to reducing the number of lesional infiltrating leukocytes, montelukast was also found to modulate M2 macrophage polarization, an anti-inflammatory phenotype, by upregulating the expression of arginase-1 and IL-10, two important markers of M2 macrophages [16]. Leucocytes produce proinflammatory cytokines, such as TNF-*α* and IFN-*γ*, which contribute to SMC apoptosis in cardiovascular disease [51]. The anti-inflammatory property of montelukast may also help SMCs survive PPE challenge in the current experimental AAA model.

Angiogenesis is also involved in aortic aneurysm, and CysLT1 receptors mediate endogenously regulated microvessel growth [1, 52–56]. PPE infusion also promoted vascular wall remodeling partly by boosting the inflammatory process, and a thickened aortic wall may form a hypoxic microenvironment and enhance neovascular sprouting. In this study, montelukast treatment also significantly reduced the number of mural neovessels. A previous study also revealed that montelukast reduced chlorine exposure–induced increases in IL-6 and VEGF levels [57]. Downregulation of inflammatory cytokine and VEGF expression may be responsible for the reduction in mural angiogenesis induced by montelukast treatment. Another LT receptor antagonist, zafirlukast, is reported to improve atherosclerosis by mediating lipid metabolism and preventing the formation of foam cells [58]. Its effect on AAA remains to be clear in future studies. In conclusion, montelukast treatment was found to improve PPE-induced experimental nondissecting AAAs in this study, and the mechanism may partly rely on the anti-inflammatory effects of this widely used old drug.

It should be pointed out that there were some limitations in the current study. Only the PPE-induced model was used to test the effects of montelukast treatment on AAA. The other models, such as the CaCl_2_ model, should be used to confirm the results in future studies. In fact, differential susceptibilities in the different species or mouse strains really exist when inducing AAA models [59–61]. Therefore, other rodent's AAA models are needed to confirm the current findings and may be helpful in translating to human conditions.

## Data Availability

The data that support the findings of this study are available from the corresponding author upon reasonable request.
